# Adult-onset sporadic chorea: real-world data from a single-centre retrospective study

**DOI:** 10.1007/s10072-021-05332-w

**Published:** 2021-05-26

**Authors:** Roberta Bovenzi, Matteo Conti, Rocco Cerroni, Mariangela Pierantozzi, Alessandro Stefani, Antonio Pisani, Nicola Biagio Mercuri, Tommaso Schirinzi

**Affiliations:** 1 Unit of Neurology, Department of Systems Medicine, University of Rome “Tor Vergata”, Via Montpellier, 00133 Rome, Italy; 2 Department of Brain and Behavioral Sciences, University of Pavia, Pavia, Italy; 3 IRCCS Mondino Foundation, Pavia, Italy; 4 IRCCS Fondazione Santa Lucia, European Centre for Brain Research, Rome, Italy

**Keywords:** Sporadic chorea, Athetosis, Ballism, Secondary movement disorders

## Abstract

**Background:**

Adult-onset sporadic chorea includes a wide and heterogeneous group of conditions whose differential diagnosis and treatments are often challenging and extensive.

**Objectives:**

To analyse retrospectively cases of adult-onset sporadic chorea from a single Italian centre to provide insights for a practical approach in the management of these patients.

**Methods:**

A total of 11,071 medical charts from a 9-year period (2012–2020) were reviewed, identifying 28 patients with adult-onset sporadic chorea (genetic forms excluded). All available data regarding phenomenology, diagnostic workup, aetiology, treatments, and long-term outcome from this cohort were collected and analysed.

**Results:**

Adult-onset sporadic chorea occurred more frequently in females and presented with an acute-subacute onset. Cerebrovascular diseases accounted for 68% of aetiology; further causes were structural brain lesions, internal diseases, and other movement disorder syndromes. Clinical course was mild, with spontaneous resolution or minimal disturbances in 82% of cases. Neuroimaging was fundamental to diagnose 76% of adult-onset sporadic chorea, an appropriate clinical examination contributed to the 14% of diagnoses, whereas basic laboratory tests to the 10%.

**Conclusions:**

Revision of real-world data of adult-onset sporadic chorea patients from a single Italian cohort suggests that an accurate clinical examination, neuroimaging, and routine laboratory tests are useful to identify those cases underlying potentially severe but treatable conditions. Although in the majority of cases adult-onset sporadic chorea has mild clinical course and good response to symptomatic treatments, it is essential to run a fast diagnostic workup.

## Introduction

Clinical diagnosis of chorea relies on the identification of irregular, non-stereotyped, involuntary movements that seem to flit from one body region to another, like a dance. Actually, chorea could be part of a spectrum of involuntary hyperkinesia, which also includes ballism (more proximal, violent, and rapid movements of the limbs) and athetosis (more distal, slower, “snake-like” movements) [1] [2].

Aetiology of chorea may be either genetic or acquired, with both adult and younger onset. In adults, choreic syndromes are most commonly due to Huntington’s disease (HD) or levodopa-induced dyskinesia. Aside from these two main conditions, adult-onset chorea includes rare inherited disorders with chronic-progressive course and sporadic forms secondary to vascular, structural, systemic, or drug-induced causes [3–5].

Therefore, the differential diagnosis of adult-onset sporadic chorea (AOSC) is extensive and challenging; however, an accurate and timely diagnostic definition is mandatory for cases underlying life-threatening or treatable conditions.

In this perspective, analysis of real-world data, obtained through routine clinical practice, could be particularly useful to develop approach strategies allowing an appropriate management of most frequent conditions.

Here we thus retrospectively analysed a cohort of AOSC from a single Italian centre to highlight those features that may support clinicians into the diagnostic workup and therapeutic choices.

## Methods

An AOSC cohort was collected reviewing electronic medical charts of inpatients admitted to the Neurology Unit of Tor Vergata Hospital (Rome, Italy) from January 2012 to November 2020. Patients with genetically determined chorea (n = 6, 50% female, diagnosed with HD by specific genetic test) and parkinsonism with levodopa-induced dyskinesia were excluded. For each patient, demographic and clinical data, including phenomenology, comorbidity, treatments, diagnostic investigations, and final diagnosis, were recorded. The long-term outcome has been determined by a follow-up phone call in November 2020.

Neuroimaging was reviewed applying the age-related white matter changes (ARWMC) score, which evaluates the presence of both white matter and basal ganglia lesions on computer tomography (CT) or magnetic resonance imaging (MRI) brain scans [6].

The study was performed following local ethical standards and principles of Helsinki declaration.

### Statistical analysis

Descriptive statistics was conducted on both qualitative and quantitative variables by IBM-SPSS.23.

## Results

### AOSC cohort

During the 9-year study period, 11,071 patients were admitted to the Neurology Unit of Tor Vergata Hospital and 34 of them were diagnosed with a choreic syndrome (0.3%). Six patients had genetically confirmed HD; 28 patients were instead classified as AOSC, being frequency of AOSC of one case for every 395 attendances to the Neurology Unit (0.25%). Table 1 summarizes main clinical features of the cohort.

**Table 1:** Main features of AOSC cohort (*n* number, *FMD* functional movement disorder)

Number of patients	
28	
Sex	
Female: n = 19; 68%	
Male: n = 9; 32%	
Age at onset	
65.6 years (range 19–87)	
Clinical presentation	
Hemichorea: n = 16; 57%	
Choreoathetosis: n = 7; 25%	
Ballism: n = 3; 11%	
Orofacial dyskinesia: n = 2; 7%	
Type of onset	
Acute (24–48 hours): n = 12; 43%	
Subacute (48 hours to 1 week): n = 9; 32%	
Chronic (> 1 week): n = 7; 25%	
Aetiology	
Vascular: n = 19; 68%	
Structural brain lesion: n = 2; 7%	
FMDs: n = 2; 7%	
Hyperglycaemia: n = 1; 3.5%	
Hepatic encephalopathy: n = 1; 3.5%	
Encephalitis: n = 1; 3.5%	
Drug-induced: n = 1; 3.5%	
Tic disorder: n = 1; 3.5%	
Comorbidities	
Blood hypertension: n = 14; 50%	
Diabetes mellitus: n = 9; 32%	
Mood disorders: n = 6; 21%	

### Diagnostic workup and etiological diagnosis

In 68% of cases, patients were admitted after the access to the Emergency Room (in other cases as scheduled hospitalization after clinic visit).

All patients underwent routine blood exam, including complete blood cell count, coagulation tests, kidney and liver function, electrolytes, proteinogram, fasting glucose, and lipid panel. Severe hyperglycaemia (599 mg/dl) was found in one patient, whereas mild hyperglycaemia (> 100 mg/dl) in 36% of cases. Liver insufficiency resulted in one patient. Dyslipidaemia (triglycerides > 150 mg/dl and/or total cholesterol >180 mg/dl) occurred in 50% of cases.

The 29% of patients received a basic screening for autoimmunity, including antinuclear antibodies (ANA), lupus anticoagulant titre (LAC), anti-neutrophil cytoplasmic antibodies (c-ANCA), perinuclear anti-neutrophil cytoplasmic antibodies (p-ANCA), anti-cardiolipin, and anti-beta2 glycoprotein antibodies research. LAC was positive in two patients and ANA in one.

The 11% of cases were tested for streptococcal infection by anti-streptolysin antibodies titre (all negative), and the 18% for acanthocytes presence by blood smear (all negative).

The 18% of patients received lumbar puncture for CSF analysis, which demonstrated aseptic encephalitis in one case.

All patients underwent neuroimaging (brain MRI: 93%; brain CT: 7%). Small vessel disease resulted as the most common finding, involving white matter in 82% of patients and basal ganglia in 43%. An acute ischaemic lesion resulted in twelve patients (43%). Stroke was located in the right globus pallidus (n = 2), right putamen (n = 1), right corona radiata (n = 1), right thalamus (n=1), left thalamus (n = 2), left insula (n = 1), left temporo-parieto-occipital cortex (n = 2), right frontal cortex (n = 1), and bilateral thalamus and subthalamus because of deep vein thrombosis of thalamic veins (n = 1).

Other brain MRI findings included symmetrical T1 hyperintensities in both pallidal nuclei suggestive of hepatic encephalopathy (n = 1), severe ventricular enlargement suggestive of normal pressure hydrocephalus (n = 1), and T2 FLAIR hyperintensities with T1 post-gadolinium enhancement into the right thalamus, midbrain, caudate, and periventricular areas suggestive of brain lymphoma (n = 1).

**Fig. 1:**
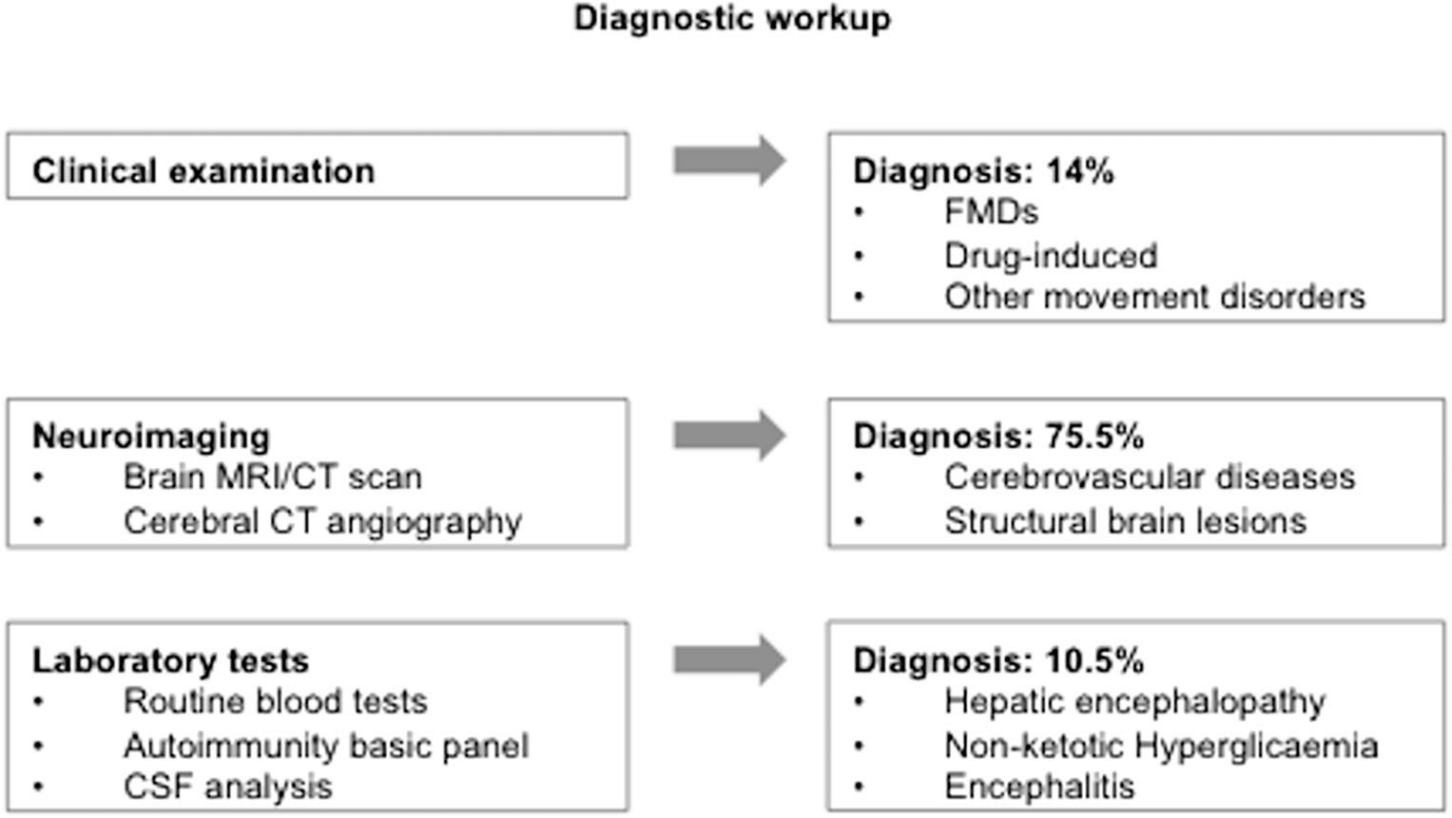
Schematic representation of the diagnostic workup with the contribution of single investigations to the final diagnosis of AOSC

Cerebral CT angiography was performed in two patients and showed in both cases a sub-occlusive stenosis of the left internal carotid (Fig. 1).

### Treatments and outcome

The 32% (n = 9) of patients had transient disturbances that spontaneously finished within few days after onset (< 1 week).

In the remaining 19 patients, a symptomatic treatment was administered with benefit in six cases (haloperidol: n = 4; tetrabenazine: n = 1; diazepam: n = 1).

An etiological treatment was instead approached in three cases, namely the normal pressure hydrocephalus by a shunt placement, the aseptic encephalitis by antiviral therapy, and the hepatic encephalopathy by cathartic enemas. In all these cases, chorea completely remitted following the interventions.

Antiplatelet/anticoagulant therapy has been set in all cases of ischemic origin, whereas antidiabetic, lipid-lowering, and antidepressants have been set to treat most frequent comorbidities.

At long-term follow-up, chorea was recovered in 18 patients (64.3%); in five cases (18%), involuntary movements persisted but were tolerated without treatments. Four patients were lost at follow-up; one patient, that one diagnosed with bilateral thalamic venous infarction, died during hospitalization.

## Discussion

This retrospective study aimed at reviewing real-world data from a single Italian centre about the clinical management of AOSC, in order to highlight those features that may support appropriate diagnosis and treatments of a heterogeneous condition due to a variety of causes [3].

In our experience, frequency of AOSC was of one case every 395 inpatients. Consequently, into a 9-year period, a total of 28 patients were collected.

In our centre, AOSC mostly involved late adulthood (mean age of onset 65.6 years). Females were more affected (68%) than males. Onset was mostly acute or subacute (75%), consistently with the main aetiology, which was vascular in almost 70% of cases, as other authors previously reported [7].

In facts, chorea may represent either a manifestation of acute ischaemic stroke [8, 9] or a post-stroke motor complication [10], especially when lesions interest basal ganglia, thalamus [10] [11], and cortex [12] [13]. In our population, we observed nine AOSCs due to acute ischaemic stroke; two patients with a “limb shaking” transient ischaemic attack [14], namely hemichorea-like movements due to sub-occlusive stenosis of internal carotid; and one patient (male, 64 years old) with athetosis, mental slowness, and speech disturbances due to the thrombosis of thalamic veins with bilateral thalamic infarction, a condition that has been already reported to provoke blepharospasm [15] and choreoathetosis [16]. In seven patients, instead, AOSC was associated with chronic vascular encephalopathy.

Neuroimaging, which actually was performed in all patients here enrolled, was thus fundamental to diagnose such cases of vascular origin. However, neuroimaging also allowed diagnosing those cases due to structural brain lesions, specifically the primary CNS lymphoma (PCNSL) and the idiopathic normal pressure hydrocephalus (iNPH).

Both these conditions are very rare causes of chorea, but some report already exists. In fact, PCNSL, because of the preferential localization into the basal ganglia [17], may cause movement disorders [18]. Here, the patient with PCNSL (female, 57 years old), recently described by Grillo [18], developed subacute choreoathetosis in left hand associated to the neoplastic infiltration of thalamus, caudate, midbrain, and periventricular regions of the right hemisphere and significantly ameliorated after mass reduction. As well, chorea may complete the clinical spectrum of iNPH, resulting from the dynamic stress of subcortical motor circuits [19]. In our case, subacute choreoathetosis and oromandibular dyskinesia occurred in one patient (female, 56 years old) fulfilling diagnostic criteria for iNPH and completely remitted after shunt placement.

Diagnoses of 10.7% AOSCs were supported by laboratory tests on biofluids, in particular those due to hyperglycaemia, antiphospholipid antibodies syndrome, hepatic insufficiency, and aseptic encephalitis.

It is well known that acute hemichorea/hemiballism with typical neuroimaging abnormalities in the striatum can complicate episodic non-ketotic hyperglycaemia in diabetic patients [20, 21]. In our patient (female, 87 years old), acute hemichorea occurred during a hyperglycaemic peak (599 mg/dl) and completely recovered in few days by antidiabetic treatment and haloperidol administration.

In antiphospholipid antibody syndrome (APS), chorea may rarely occur because of the antibody-mediated inflammation of basal ganglia [22]. Here, we had a patient (female, 69 years old), suffering with APS [23], that presented left athetosis associated with right frontal ischaemic stroke. In addition, we found high titres of LAC and ANA in two other patients with chorea of vascular origin, suggesting that such autoantibodies could have a more complex role in pathophysiology of chorea, which probably includes some vascular mechanism [24]. Overall, in these three patients, chorea had a self-remitting course.

In another case, instead, we had a patient (male, 64 years old), affected by alcohol-related cirrhosis, who developed subacute generalized chorea and orofacial dyskinesia during an episode of liver insufficiency, progressively resolved by cathartic enemas. A subsequent brain MRI showed symmetrical abnormalities into the globus pallidus, as typically observed in hepatic encephalopathy [25]. Actually, chorea and many different movement disorders can appear in patients with hepatic encephalopathy and chronic liver diseases [26]. As well, chorea and other movement disorders have been reported early, in the acute phases, or later, as a delayed effect of the infection, in encephalitis due to HIV, influenza virus, flavivirus, and herpesvirus [27]. In our case of aseptic encephalitis (extensive neurotropic virus screening resulted negative), subacute chorea evolved into a complex condition, including speech disturbances and mental slowness, which completely disappeared at the resolution of brain inflammation, after antiviral therapy (acyclovir).

In four cases (14%), our diagnoses were purely clinically based. Two patients had functional movement disorders (FMDs), diagnosed by recently proposed criteria [28]*.* The first one (female, 67 years old), with orofacial dyskinesia, was relative of a genetically confirmed HD case, a condition particularly predisposing to FMDs [29]. The second one (female, 27 years old) had a form of subacute functional chorea associated with post-traumatic stress disorder (PTSD), which is often comorbid to FMDs [30]. Another patient (female, 52 years old) had a typical tardive syndrome [31], with facial/tongue dyskinesia and choreoathetosis following a 4-year story of neuroleptics and mood stabilizers administration. Last patient (male, 19 years old), presenting with acute, proximal ballistic-like limb movements associated with more distal dyskinesia, was finally diagnosed with tic disorder, consistently with his previous history. In fact, phenomenology of tics is highly variable over lifespan, mimicking other movement disorders, such as myoclonus, dystonia, or chorea [32].

Although heterogeneous, this group of AOSC was characterized by essentially mild clinical course, with spontaneous resolution, or minimal and tolerated disturbances, in 82% of cases*.* When necessary, symptomatic therapy (mostly haloperidol) was effective; as well, etiological treatments in specific conditions favoured full recovery of motor symptoms.

This study has several limitations, including the sample size and the retrospective design. In addition, since we reviewed only inpatients of a Neurology Unit, we missed cases from other units that might have faced with rapid and transient episodes of movement disorders (such as those drug induced) or forms due to metabolic diseases, thus underestimating the overall frequency of AOSC and the prevalence of such aetiologies.

Despite such limitations, real-world experience indicated that sporadic chorea in late adulthood mostly affects females, opening a speculative question about a potential gender-related vulnerability. Nevertheless, acute or subacute onset and vascular aetiology are definitely the most frequent. Consequently, early neuroimaging is fundamental to diagnose those cases that, although rare, may underlie severe and life-threating conditions amenable to time-dependent treatments (e.g. carotid occlusion or cerebral vein thrombosis). Likewise, neuroimaging allows identifying cases due to structural brain lesions, which may benefit of dedicate interventions.

In the diagnostic process, routine blood tests investigate vascular risk factors and metabolic alterations, which can cause independently movement disorders (e.g. hepatic insufficiency, hyperglycaemia). A basic autoimmunity panel is also useful to individuate the antibodies that may cause chorea directly, by immune-mediate mechanisms, or indirectly, through a cerebrovascular disease. On the other hand, research of acanthocytes and anti-streptococcal antibodies deserved specific presentations. CSF analysis instead confirms cases of encephalitis in which chorea is part of a more complex and suggestive syndrome.

Adequate anamnesis recording and clinical examination remain crucial to diagnose either drug-induced and functional conditions or other hyperkinetic movement disorders that may be confused with chorea and ballism.

Finally, both the substantial benign course of most of AOSCs and the good response to symptomatic and etiological treatments should inspire the approach to the management of these patients.
